# Hesperetin Protects from Palmitic-Acid-Induced Lipotoxicity through the Inhibition of Glutaminolysis, mTORC1 Signaling, and Limited Apoptosis

**DOI:** 10.1021/acs.jafc.5c05570

**Published:** 2025-08-21

**Authors:** Wan Li, Zhengnan Cai, Florian Schindler, Martin Brenner, Christian Winter, Bianca Stiller, Petra Heffeter, Wolfram Weckwerth

**Affiliations:** † Molecular Systems Biology (MOSYS), Department of Functional and Evolutionary Ecology, 27258 University of Vienna, Vienna 1030, Austria; ‡ Vienna Doctoral School of Ecology and Evolution, 27258 University of Vienna, Vienna 1030, Austria; § Vienna Doctoral School of Pharmaceutical, Nutritional and Sports Sciences, 27258 University of Vienna, Vienna 1090, Austria; ∥ Marine Biology, Department of Functional and Evolutionary Ecology, 27258 University of Vienna, Vienna 1030, Austria; ⊥ Center for Cancer Research and Comprehensive Cancer Center, 27271 Medical University of Vienna, Vienna 1090, Austria; # Research Cluster “Translational Cancer Therapy Research”, 27258 University of Vienna, Vienna 1090, Austria; ∇ Vienna Metabolomics Center (VIME), 27258 University of Vienna, Vienna 1030, Austria; ○ Health in Society Research Network, 27258 University of Vienna, Vienna 1030, Austria

**Keywords:** hesperetin, lipotoxicity, glutaminolysis, mTORC1, isotope labeling

## Abstract

Palmitic acid-induced lipotoxicity contributes to the development of nonalcoholic fatty liver disease (NAFLD). Hesperetin has been reported to alleviate oxidative stress, inflammation, and cell death in NAFLD, while its potential to mitigate palmitic acid-induced lipotoxicity remains unexplored. This study investigates the protective effects of hesperetin on palmitic-acid-stimulated lipotoxicity and elucidates the underlying molecular mechanisms. Our results showed that hesperetin decreased palmitic acid-activated lipotoxicity through inhibition of the intrinsic apoptosis pathway and promotion of autophagic flux. Metabolomics analysis and stable-isotope-tracing data indicated that hesperetin treatment restored the aberrant tricarboxylic acid cycle caused by palmitic acid exposure, accompanied by a decrease in anaplerotic flux from glutamine to α-ketoglutarate. The reduction of α-ketoglutarate resulted in the inhibition of mTORC1 signaling, which in turn activated autophagy and limited apoptosis. Furthermore, hesperetin activated AMPK, which coordinated with mTORC1 to regulate autophagy. Additionally, hesperetin reinstated the activation of AKT and Nrf2, further protecting the cell against the deleterious effects of lipotoxicity. These data highlight the role of glutaminolysis as a survival mechanism for preventing lipotoxicity upon hesperetin treatment.

## Graphical abstract

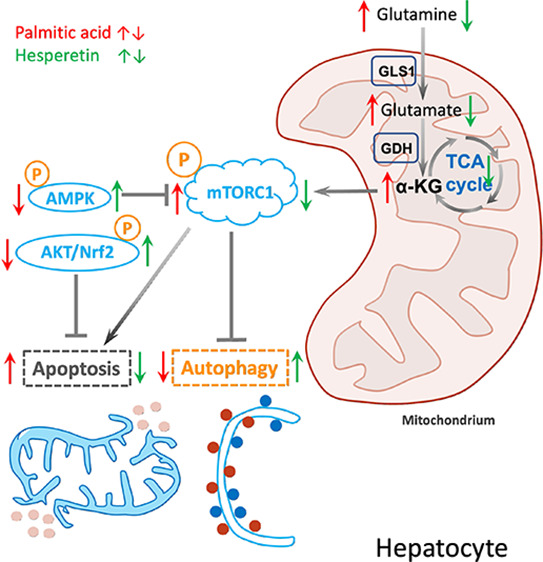

## Introduction

The elevated concentrations of free fatty acids (FFAs) within the liver have been recognized as a characteristic of nonalcoholic fatty liver disease (NAFLD), the most common complication of diabetes and metabolic syndrome.^1^ Lipid overload exceeds the metabolic capacity of hepatocytes to dispose of FFAs, promoting the generation of lipotoxic intermediates.^2^ Among these FFAs, palmitic acid (PA, C16:0) exerts greater hepatotoxicity than unsaturated fatty acids, such as oleic acid (OA, C18:1), primarily through the activation of apoptotic pathways and subsequent hepatocellular dysfunction. This phenomenon is known as lipotoxicity or lipoapoptosis.^2,3^ PA-induced lipotoxicity, driving a sustained tissue damage and promoting fibrogenesis, is believed to be a key initiating event in the pathogenesis of NAFLD.^2,4^ The mechanisms underpinning PA-triggered lipotoxicity have been extensively investigated and have revealed several pathways that govern the toxic effects of PA in hepatocytes.^2,5,6^ Recent evidence has demonstrated that macroautophagy (autophagy) impairment plays a pivotal role in PA-induced lipotoxicity.^7^ Autophagy is a highly conserved, lysosome-dependent catabolic process responsible for the degradation of intracellular components.^8^ Impaired autophagic activities fail to regulate physiological functions, leading to various pathological conditions. This can be largely attributed to its role in the removal of damaged organelles, such as mitochondria and endoplasmic reticulum (ER), as well as its contribution to the clearance of lipid accumulation through lipophagy.^9^ In vivo and in vitro data provide compelling evidence that targeting defects in hepatic autophagy could effectively prevent lipid accumulation and hepatocellular dysfunction.^10,11^ The process of autophagy is modulated by a number of signaling molecules; mammalian TORC1 (target of rapamycin complex 1) (mTORC1) is one of the most important components that negatively regulate autophagy at both the initiation and completion stages. mTORC1 could integrate fluctuations in the availability of nutrients to orchestrate the autophagic machinery and, therefore, maintain cellular and organismal homeostasis and function.^12,13^ The S6 ribosomal protein (S6) and eIF4E-Binding Protein 1 (4E-BP-1) are key targets of mTORC1, and their phosphorylation represents a downstream event in the mTORC1 signaling pathway, commonly used as markers to assess mTORC1 activation.^13,14^ AMP-activated protein kinase (AMPK) was initially described as a cellular energy sensor that is critical for the maintenance of cellular energy homeostasis. AMPK can directly activate or inhibit its downstream effector proteins, thereby coordinating cellular adaptive responses to metabolic and environmental stresses.^15^ A substantial body of evidence has highlighted the involvement of AMPK in the regulation of autophagy and apoptosis, with its action linked to the negative modulation of the mTORC1 pathway.^15,16^ Furthermore, protein kinase B (AKT) and nuclear factor erythroid 2-related factor 2 (Nrf2) have been suggested to play crucial and interconnected roles in the modulation of FFA-triggered dysfunction.^17,18^ AKT activation regulates lipid metabolic pathways that control lipid storage, utilization, and lipotoxicity,^19,20^ while Nrf2 activation triggers the expression of antioxidant enzymes, like heme oxygenase 1 (HO-1), protecting cells from oxidative stress-induced cell apoptosis.^21^

Glutamine, the most abundant amino acid in the plasma, undergoes enzymatic reactions, namely glutaminolysis, after being transported into cells.^22^ Glutaminolysis comprises two steps, whereby glutamine is first hydrolyzed to glutamate by glutaminase (GLS), and then the generated glutamate is converted to α-ketoglutarate (α-KG) by glutamate dehydrogenase (GDH).^22,23^ Glutamine-derived α-KG replenishes the tricarboxylic acid (TCA) cycle for ATP synthesis and other key metabolic intermediates for biosynthesis of proteins, lipids, and nucleotides.^24^ Glutaminolysis is recognized as a crucial process for tumor growth and is frequently found to be upregulated in various types of cancers.^22,25^ Recent studies have highlighted the tight association of dysregulation of glutaminolysis with the development of metabolic diseases, such as aging-related disorders,^26^ heart injury,^27^ liver fibrosis,^28^ diabetes mellitus, and nonalcoholic steatohepatitis (NASH).^29^ In particular, Glutaminolysis was found to contribute to PA-induced lipotoxicity by elevating TCA flux and oxidative stress.^6^ Interestingly, it is proposed that this metabolic reprogramming promotes mTORC1 activation through the Rag GTPases, leading to the subsequent inhibition of autophagy.^30^ Additionally, the association of glutaminolysis and mTORC1 mediates the induction of apoptosis linked to the disruption of autophagic flux under conditions of nutrient limitation.^14^

Hesperidin (Hsd β-7-rutinoside of hesperetin) is a natural compound predominantly found in citrus fruits, such as lemons, oranges, limes, and grapefruits.^31^ Upon ingestion, Hsd undergoes enzymatic hydrolysis in the intestine, resulting in its conversion to the aglycone form hesperetin (Hst) (Figure 1). Hst is considered to be directly absorbed in the intestine.^32^ Hst, a major active ingredient of traditional Chinese medicine chenpi,^18^ has attracted increasing attention for its diverse pharmacological activities.^33,34^ Previous studies reported that Hst exhibits promising protective effects against NAFLD. Mechanistically, Hst has been demonstrated to be capable of inhibiting OA-induced oxidative stress and inflammation in hepatocytes, as a consequence, mitigating the progression of NAFLD.^18^ However, the mechanisms that contribute to its hepatoprotective effects are still not completely understood. It is well accepted that unbalanced glutaminolysis is closely associated with NAFLD.^6,28^ Besides, induction of glutaminolysis is linked to activation of the mTORC1 pathway, which contributes to hepatocellular lipotoxicity.^6,35^ Therefore, in this present work, we set out to elucidate the factors involved in mediating the hepatoprotective effects of Hst against PA-induced lipotoxicity in hepatocytes.

**Figure 1:**
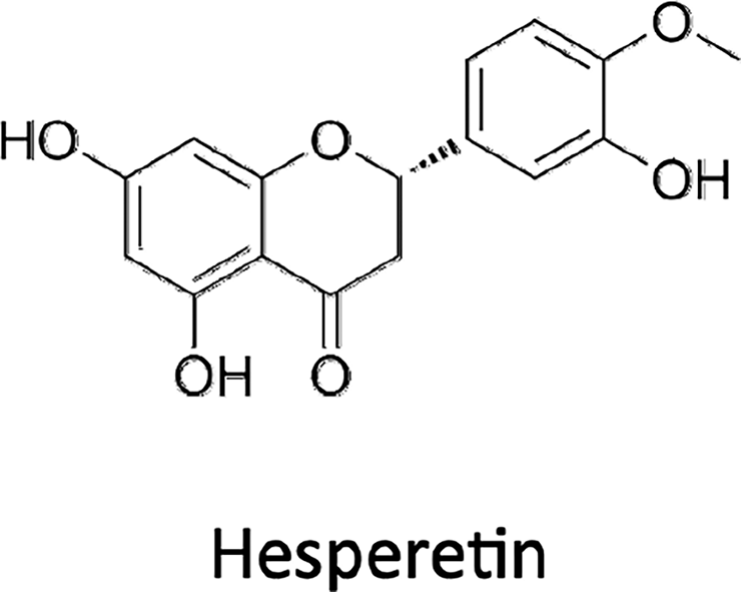
Structure of hesperetin.

## Materials and Methods

### Materials and Reagents

Dulbecco’s Modified Eagle’s Medium (DMEM) (31053028), trypsin-EDTA (15400054), fetal bovine serum (FBS) (16000044), l-glutamine (25030149), penicillin/streptomycin solution (15070063), pyruvate (11360070), dialyzed FBS (A33820-01), and mitochondrial isolation kit (89874) were purchased from Thermo Fisher Scientific (Waltham, MA, USA). U-^13^C-glutamine (CLM-1822-H) was provided by Cambridge Isotope Laboratories (Tewksbury, MA, USA). 4′,6-Diamidino-2-phenylindole (DAPI, MBD0015), chloroquine (CQ, C6628), and palmitic acid (PA, P0500) were obtained from Sigma-Aldrich (St Lois, MO, USA). FITC Annexin V Apoptosis Detection Kit with 7-aminoactinomycin D­(7-AAD, 640922) was bought from BioLegend (Amsterdam, The Netherlands). Dimethyl-α-ketoglutarate (DMKG, sc-211344) and hesperetin (Hst, sc-252878) were obtained from Santa Cruz Biotechnology (Heidelberg, Germany), and bis-2-(5-phenylacetamido-1,2,4-thiadiazol-2yl)­ethyl sulfide (BPTES) (HY-12683) and Rapamycin (RAP) (HY-10219) were purchased from MedChemExpress (Monmouth Junction, NJ, USA). All other reagents were of analytical grade and were from Sigma Chemicals Co. (St Lois, MO, USA).

### Preparation of PA and Hesperetin

Briefly, PA was dissolved in 0.1 M NaOH solution at 70 °C until completely solubilized and then mixed with 10% fatty acid-free BSA at 40 °C. Next, the PA solution was filtered through a 0.22 μm filter and stored at −80 °C.

A 100 mM Hst stock solution in DMSO was prepared and kept at −80 °C.

### Cell Culture and Treatments

HepG2 cells were obtained from the American Type Culture Collection (ATCC, Manassas, VA) and cultured in DMEM containing 10% heat-inactivated FBS, 2 mM l-glutamine, 100 U/mL penicillin, and 100 μg/mL streptomycin. For the metabolomic analysis, FBS was replaced with dialyzed heat-inactivated FBS. In the experiment, HepG2 cells were pretreated with different concentrations of Hst for 4 h and then stimulated with PA for another 10 h. In the experiment with inhibitors, BPTES, RAP, DMKG, and CQ were pretreated with HepG2 cells at final concentrations of 30 μM, 200 nM, 1 mM, and 10 μM, respectively.

### Cell Apoptosis

The apoptotic rate of HepG2 cells was analyzed by flow cytometry using annexin V and 7-AAD staining. HepG2 cells were seeded into a 6-well plate at a density of 1 × 10^5^ cells/mL and incubated with treatments for 10 h. Afterward, the cells were washed with cold washing buffer (420201, BioLegend, The Netherlands) three times and were suspended in annexin V-binding buffer, followed by incubation with FITC-annexin V and 7-AAD for 10 min in the dark. The apoptosis of cells was measured by FACSAria llu (BD Bioscience, New York, USA).

### Immunofluorescence Staining

1.8 × 10^5^ cells of HepG2 were grown in confocal dishes overnight and received the above-mentioned treatments. Thereafter, the cells were fixed with cold 4% paraformaldehyde (PFA) in phosphate-buffered saline (PBS) (T61899-AK, VWR, Austria) for 20 min at room temperature and were permeabilized with 0.5% Triton X-100 in PBS for 5 min. 1% bovine serum albumin (BSA) in PBS was used to block nonspecific sites. Then the cells were incubated with primary antibodies (anti-*p*-ribosomal protein S6, dilution 1:200 or anti-LC3α/β dilution 1:200) at 4 °C overnight. After washing three times with tris-buffered saline containing Tween 20 (TBST), the cells were treated with the corresponding secondary antibody (Coralite 488-conjugated goat antimouse IgG (H+L) (SA00013-1, dilution 1:500). Finally, DAPI (1:400) was used for nuclear counterstaining. Images were taken on a confocal laser scanning microscope (LSM700, Carl Zeiss, Germany) and processed by the Zeiss ZEN2.5 software.

### Mitochondrial Preparation

A mitochondrial isolation kit was used to isolate the mitochondrial fractions. Briefly, 24 × 10^6^ HepG2 cells were seeded into 150 mm dishes. Upon treatments, the cells were suspended in 800 μL of reagent A containing protease inhibitor cocktail (4693116001, Sigma, USA) for 2 min on ice. Then 10 μL of reagent B was added and incubated for 5 min. Afterward, the reactions were set up with 800 μL of reagent C. Subsequently, 10 μL of Reagent B was added, followed by a 5 min incubation. The mixture was then set up with 800 μL of Reagent C and centrifuged at 700*g* for 10 min at 4 °C. The resulting supernatant was collected and further centrifuged at 12,000*g* for 15 min at 4 °C to isolate the cytosolic fraction. The precipitate was suspended in the 500 μL of reagent C and further centrifuged at 12,000*g* for 5 min at 4 °C to pellet mitochondria. The mitochondrial pellet was lysed with 2% CHAPS in TBS, and mitochondrial protein concentration was determined using a BCA kit (23225, Thermo Fisher, USA).

### Immunoblotting

HepG2 cells were lysed using cold RIPA lysis buffer with protease inhibitor cocktail and centrifuged at 15,000*g* for 15 min at 4 °C. The supernatants were kept, and the quantification of proteins was performed using a BCA kit (71285-3, Millipore, Germany) for quantification. Equal amounts of proteins were separated by 10–12.5% SDS PAGE and were transferred onto polyvinylidenfluoride (PVDF) membranes (Millipore, Darmstadt, Germany). The membranes were blocked with 5% no-fat milk (sc-2324, Santa Cruz Biotechnology, Germany) at room temperature for 1h and then incubated with primary antibodies at 4 °C overnight. After washing three times with TBST, the membranes were treated with peroxidase-labeling secondary antibodies for 1 h. The protein bands were detected by the ECL system (WBKLS0100, Millipore) and acquired using the iBright FL1500 Imaging System (Invitrogen, Carlsbad, USA). The signal intensity was quantified by ImageJ. The antibodies mentioned were anticaspase-3 p17 (sc-271028), anti-LC3α/β (sc-398822), anticytochrome C (sc-13156), anti-Bax (sc-7480), anti-Bcl-2 (sc-7382), anti-p-ribosomal protein S6 (sc-293144), antiribosomal protein S6 (sc-74459), anti-p-4E-BP-1 (sc-293124), anti-4E-BP-1 (sc-81149), anti-AMPK (sc-74461), and antiheme oxygenase-1 (HO-1, sc-136960) were purchased from Santa Cruz (Heidelberg, Germany). Anti-p62 (5114), anti-p-AMPK (Thr172) (2535), anti-p-Akt (Ser473) (4060), and Akt (4691) were obtained from Cell Signaling (Danvers, USA). Anti-α-tubulin (1224-1-AP), anti-Tom20 (11802-1-AP), horseradish-peroxidase (HRP)-conjugated anti-Rabbit IgG (H + L) (SA00001-2) and (HRP)-conjugated antimouse IgG (H + L) (SA00001-1) were obtained from Proteintech Group (Munich, Germany).

### Isotope-Labeling Analysis

Stable isotope tracing experiments were performed as described previously with some modifications.^36,37^ Briefly, 1 × 10^6^ HepG2 cells per mL were seeded into a six-well plate and cultured overnight. Then cells were preincubated with Hst for 4 h. Afterward, the cells were washed three times with PBS and switched to DMEM medium supplemented with 10% dialyzed FBS. One mM U-[^13^C]-glutamine, Hst, and PA were added as indicated. After treatments, cells were washed twice with ice-cooled 0.9% NaCl and quenched by adding 1 mL of 50% precooled methanol (−80 °C) containing 2.5 nM phenyl β-d-glucopyranoside (Sigma, USA) as an internal control. Cell lysates were collected in polypropylene tubes by scraping, followed by the addition of 200 μL of chloroform. The samples were shaken for 1 h at 4 °C. After centrifugation, the supernatant was transferred to a new tube and dried in a SpeedVac (Labogene, Denmark). Next, 15 μL of methoxyamine hydrochloride solution (40 mg/mL in pyridine) was added to the dried fraction, and the mixture was then incubated for 90 min at 30 °C. Subsequently, 60 μL of *N*-methyl-*N*-trimethylsilyltrifluoracetamide (MSTFA) was added and incubated for 30 min at 37 °C. The reaction mixtures were centrifuged for 10 min and 4 °C at 21,000*g*, and the supernatants were transferred to glass vials with microinserts. Measurement of metabolites was performed using GC-MS based on the standard protocols.^38^ Data processing and natural ^13^C labeling correction were performed using the Data Extraction for Stable Isotope-labeled metabolites (DExSI) software.^39^ Default settings were used, except for the following parameters: points on either side of the apex and scan window were set to 10. Mass isotopomer fraction labeling was determined by integrating metabolite ion fragments (Table 1).

**Table 1:** Metabolite-Specific Mass Fragments for the Calculation of ^13^C-Isotope Incorporation

compound	derivate	ions	formula	unlabeled	labeling with
α-KG	1MeOX 2TMS	304, 305, 306, 307, 308, 309	C11H22O5N1Si2	304	U-[^13^C]-glutamine
malate	3TMS	233, 234, 235, 236	C9H21O3Si2	233	U-[^13^C]-glutamine
citrate	4TMS	273, 274, 275, 276, 277	C11H21O4Si2	273	U-[^13^C]-glutamine
glutamate	3TMS	246, 247, 248, 249, 250	C10H24N1O2Si2	246	U-[^13^C]-glutamine

### Metabolomic Analysis by GC-MS

Cellular metabolites were extracted and analyzed using Agilent 6890 gas chromatography coupled to a LECO Pegasus 4D TOF spectrometry (GC-TOF-MS) according to previously established method with modifications.^37^ In brief, HepG2 cells were seeded into a 6-well plate at a density of 1 × 10^6^ cells/mL. The day after, cells were preincubated with Hst for 4 h, followed by exposure to PA for another 4 h. Then, cells were washed three times with precooled 0.9% NaCl and quenched by the addition of 80% methanol (−80 °C) containing 2.5 nM phenyl β-d-glucopyranoside as an internal standard. Extraction samples were incubated for 15 min at 4 °C and then centrifuged for 10 min at 21,000 *g*. The supernatant was transferred to a fresh polypropylene tube and dried in a SpeedVac. The cell pellets were lysed with RIPA and used to measure protein levels for normalization purposes. Sample derivatization was carried out as described above. The injection volume of each sample was 1 μL, and they were injected at a 1:5 split ratio. The total ion chromatogram was deconvoluted, and peak alignment and integration were performed using the software MS-DIAL.^40^

### Statistical Analysis

The results are repeated in at least three independent experiments and presented as a mean ± SEM. All statistical analyses were performed using Prism v9 (GraphPad Software) or Excel (Microsoft). Statistical significance was evaluated using an unpaired Student’s *t*-test. Differences were considered statistically significant when *p* < 0.05.

## Results

### Hst Attenuated PA-Induced Cell Apoptosis in HepG2 Cells

Our previous study demonstrated that 400 μM PA induced a 50% reduction in cell viability in HepG2 cells. However, treatment with Hst effectively prevented PA-induced cell death by restoring mitochondrial function. This protective effect was observed at both 20 and 40 μM of Hst, with a greater effect seen at 40 μM of Hst.^41^ In contrast, Hst at 10 μM did not produce a detectable effect on PA-induced apoptosis in HepG2 cells. Furthermore, our previous findings indicated that exposure to 80 μM Hst resulted in significant cytotoxicity. Since 20 and 40 μM Hst exhibited potent activity with minimal cytotoxic effects, these concentrations were selected for further evaluation. We then investigated whether the observed protection against cell death is linked to a reduction in the intrinsic apoptotic pathway. For this aim, we determined apoptotic cells using the double-positive annexin V/7-AAD staining, analyzed by a flow cytometer. As seen in Figure 2A, B, Hst treatment decreased the apoptotic population compared to PA-treated cells. On average, the apoptotic cells in HepG2 cells incubated with Hst at the concentrations of 20 and 40 μM were reduced to 20% and 16%, respectively, relative to cells treated with PA. We also detected the protein expression of the apoptotic markers cleaved caspase-3, pro-apoptotic proteins Bax and cytochrome *C* (Cyt *C*), as well as antiapoptotic protein Bcl-2 by Western blot. Likewise, we observed that Hst treatment inhibited the expression of cleavage of caspase-3, Cyt *C*, and Bax levels, while enhancing the Bcl-2 level caused by PA stimulation in HepG2 cells (Figure 2C–H). To obtain direct evidence of the mechanism by which Hst prevented PA-induced apoptosis, we analyzed the expression of Bax and Cyt *C* in both the mitochondrial and cytosolic fractions. As shown in Figure 3, PA treatment increased mitochondrial Bax levels and cytosolic Cyt *C* levels while decreasing mitochondrial Cyt *C* levels. Hst treatment was found to inhibit the release of Cyt *C* from mitochondria into the cytosol and reduce the translocation of Bax to the mitochondria. These results suggested that Hst treatment suppressed PA-induced intrinsic pathway of apoptosis through Bax-mediated release of Cyt *C* from the mitochondria, which in turn activated caspase-3, thereby decreasing cell death and increasing cell viability.

**Figure 2:**
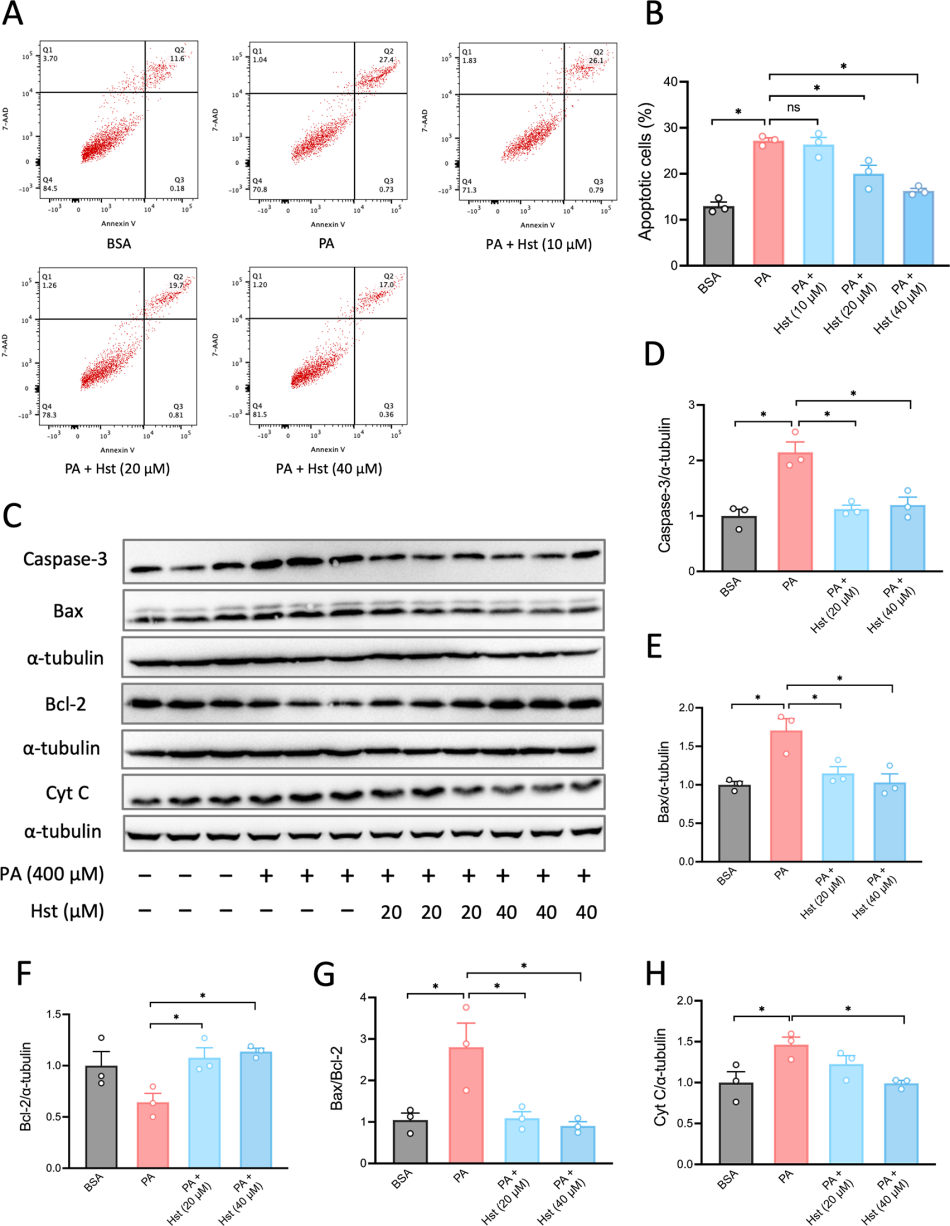
Hesperetin inhibited palmitic acid-induced apoptosis. HepG2 cells were pretreated with hesperetin (10, 20, and 40 μM) for 4 h, followed by incubation with palmitic acid (400 μM) for 10 h. Representative results of annexin V/7-AAD staining of HepG2 cells by flow cytometry were shown in (A). (B) Quantification of apoptosis population of HepG2 cells as obtained in (A). (C) Representative images of pro-apoptotic markers (cleaved Caspase-3, Bax and Cytochrome *C*) and antiapoptotic marker (Bcl-2) expressed in HepG2 cells preincubated with hesperetin (20 and 40 μM) for 4 h prior to treatment with palmitic acid (400 μM) for 10 h. (D–H) Quantification of protein expression as shown in (C). All data are presented as the mean ± SEM (*n* = 3). Two-tailed unpaired Student’s test was used to calculate statistical significance. *p < 0.05.

**Figure 3:**
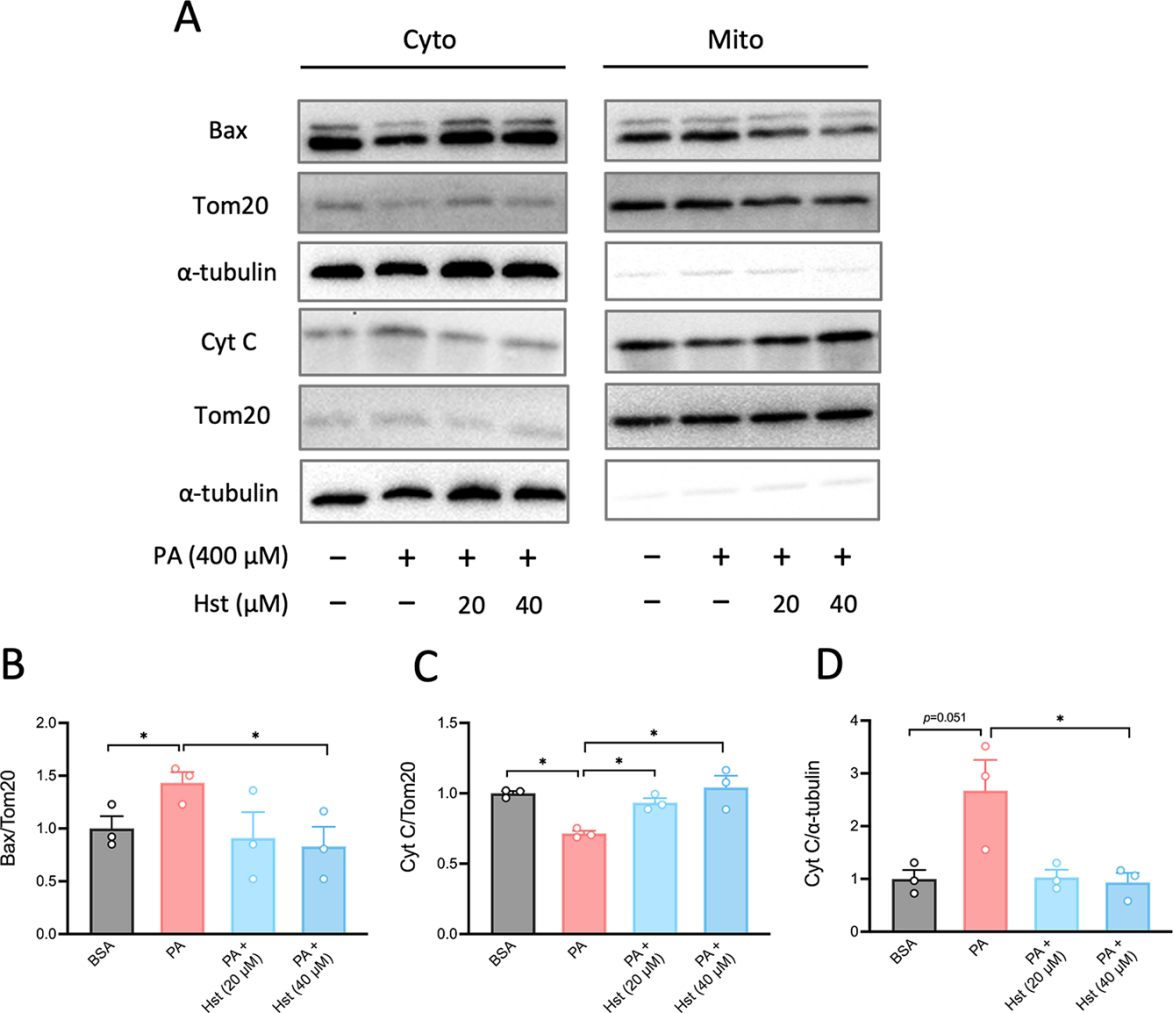
Hesperetin suppressed palmitic acid-induced intrinsic apoptotic pathway. HepG2 cells were preincubated with hesperetin (20 and 40 μM) for 4 h, then stimulated with palmitic acid (400 μM) for 10 h. (A) Immunoblot analysis of apoptotic markers (Bax and Cyt *C*) in both cytosolic and mitochondrial fractions in HepG2 cells. (B–D) Quantification of the protein expression of (A). All data are presented as the mean ± SEM (*n* = 3). Two-tailed unpaired Student’s test was used to calculate statistical significance. *p < 0.05.

### Hst Stimulated Autophagy in PA-Treated HepG2 Cells

We then investigated the molecular mechanisms through which reduced cell apoptosis in PA-treated HepG2 cells occurred. As autophagy can function as a pro-survival strategy in conditions of FFAs overload,^35^ we hypothesized that the protection of Hst against PA-induced cell apoptosis was correlated with the induction of autophagic flux. To assess the status of autophagic flux, we detected the conversion of LC3 from cytosolic form I to vesicular autophagosome-associated form II through immunoblotting and immunofluorescence. We observed that HepG2 cells incubated with Hst displayed greater numbers of punctate LC3-positive structures and enhanced steady levels of LC3-II with respect to cells in the absence of Hst after PA exposure (Figure 4A–C). In line with such results, Hst treatment was able to reduce the levels of p62 (Figure 4B, D), a selective autophagy chaperone that is utilized for detecting and delivering large biomolecules, in the presence of PA in HepG2 cells. The increase in autophagosomes might result from either the induction of autophagic flux or the inhibition of their degradation by lysosomes.^42^ We then treated HepG2 cells with chloroquine (CQ) to block the cellular degradation of autophagosomes. CQ treatment further elevated the already augmented formation of LC3-II and stimulated the reduced expression of p62 induced by Hst in PA-treated cells, indicating the induction of autophagic flux following Hst incubation (Figure 4E–G). These results indicated that Hst was sufficient to reverse the blockage in the autophagic flux impaired by PA treatment.

**Figure 4:**
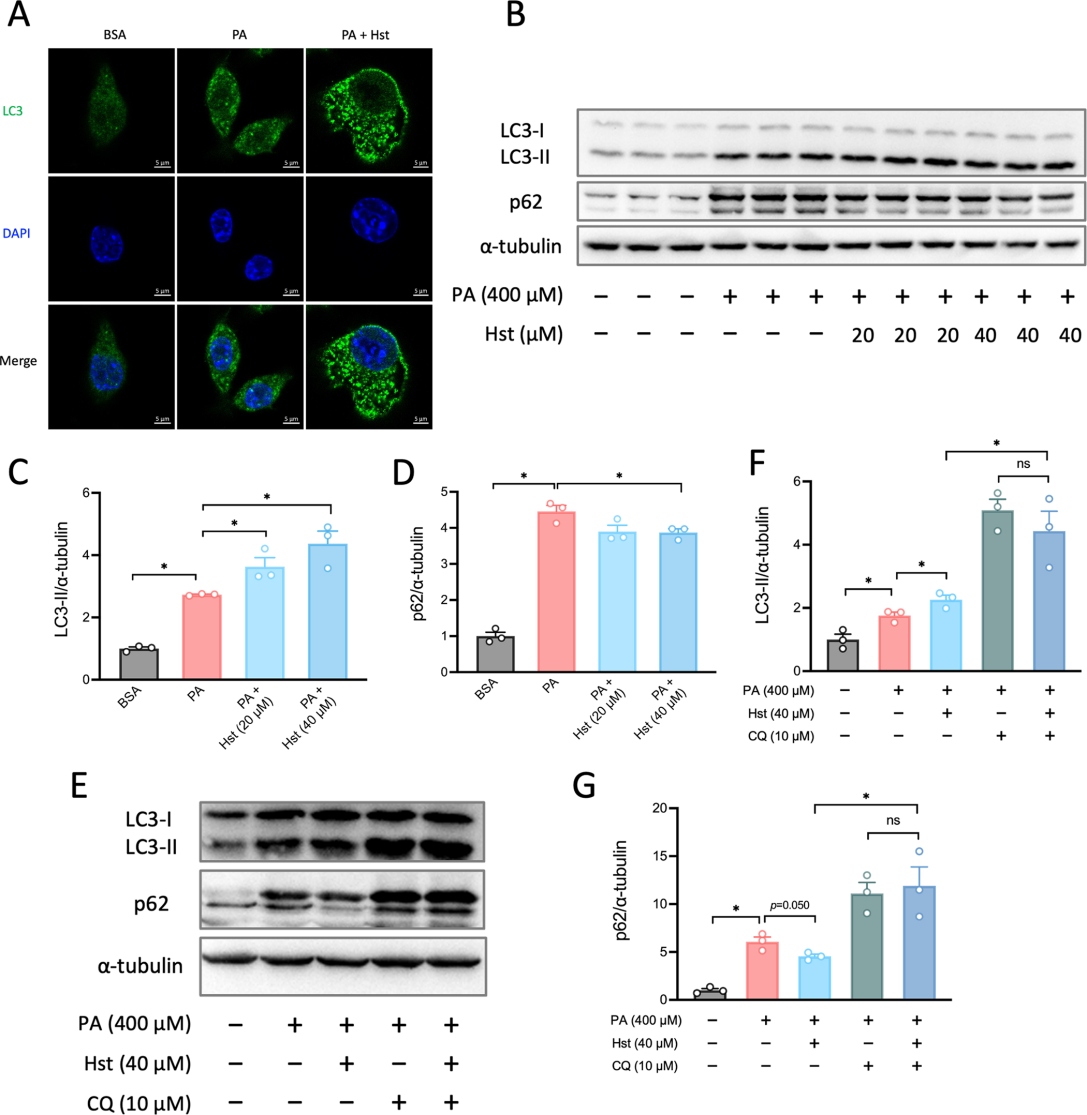
Hesperetin recovered palmitic acid-induced autophagy impairment. HepG2 cells were pretreated with hesperetin (20 and 40 μM) or chloroquine (10 μM) for 4 h, followed by incubation of palmitic acid (400 μM) for 10 h. (A) Immunofluorescence microscopy captions of HepG2 cells. Cells were stained against LC3I/II (green) and DAPI (blue). (B) Immunoblot analysis of autophagy markers (LC3I/II and p62) in HepG2 cells. (C and D) Quantification of the protein expression of (B). (E) Immunoblot analysis of autophagy markers (LC3I/II and p62) in HepG2 cells treated with CQ. (F and G) Quantification of the protein expression of (E). All data are presented as the mean ± SEM (*n* = 3). Two-tailed unpaired Student’s test was used to calculate statistical significance. *p < 0.05. Scar bars are 5 μm in (A).

### Hst Inhibited PA-Stimulated mTORC1 Activity in HepG2 Cells

To illustrate how Hst stimulated autophagy, we explored whether Hst modulated autophagy-related signaling cascades. mTORC1 complex is a well-defined upstream factor negatively regulating autophagy. As shown in Figure 5A–E, incubation of HepG2 cells with Hst was observed to be effective in dampening mTORC1 complex activity, as reflected by a decrease in the fluorescence intensity of S6 and reduced phosphorylation levels of both S6 (Figure 5A–D) and 4E-BP-1 (Figure 5C, E) in PA-stimulated HepG2 cells incubated with Hst compared to untreated cells. Furthermore, we investigated the impact of Hst on the activation of AMPK, a key upstream regulator controlling the mTORC1 complex and autophagic activities. Our results showed that AMPK activation was suppressed in PA-treated HepG2 cells compared with the control group. However, Hst treatment significantly enhanced AMPK phosphorylation (Figure 5F, G). This was paralleled by the inhibition of mTORC1 induced by Hst, consistent with the known negative correlation between AMPK activation and mTORC1 activity. We next investigated the mechanistic link between Hst-mediated suppression of mTORC1 activity and stimulation of autophagy. We found that incubation of HepG2 cells with rapamycin, the inhibitor of mTORC1, substantially enhanced the capability of Hst to inhibit mTORC1 activity (Figure 6A–C), increase LC3-II accumulation, and reduce p62 levels in PA-treated HepG2 cells (Figure 6D–F). Moreover, suppression of mTORC1 by rapamycin further downregulated the ratio of Bax/Bcl-2 in PA-stimulated HepG2 cells upon Hst incubation compared with that of the Hst-untreated counterparts (Figure 6G, H). Overall, such results demonstrated that Hst induced autophagy by regulating, at least in part, the upstream signaling cascade mTORC1 as a result of declining cell apoptosis. Clear evidence has revealed that Nrf2 is the key driver of antioxidant cascades, promoting transcription and expression of mostly antioxidant proteins, including HO-1.^21^ After 10 h treatment with Hst, HepG2 cells showed a marked increase in phosphorylation of Nrf2 (Figure S1C,D). Accordingly, Hst elevated the expression of HO-1 in PA-treated cells (Figure S1A,B). As shown in Figure S1E,F, PA impaired the activation of Akt, while Hst was observed to reactivate Akt activity.

**Figure 5:**
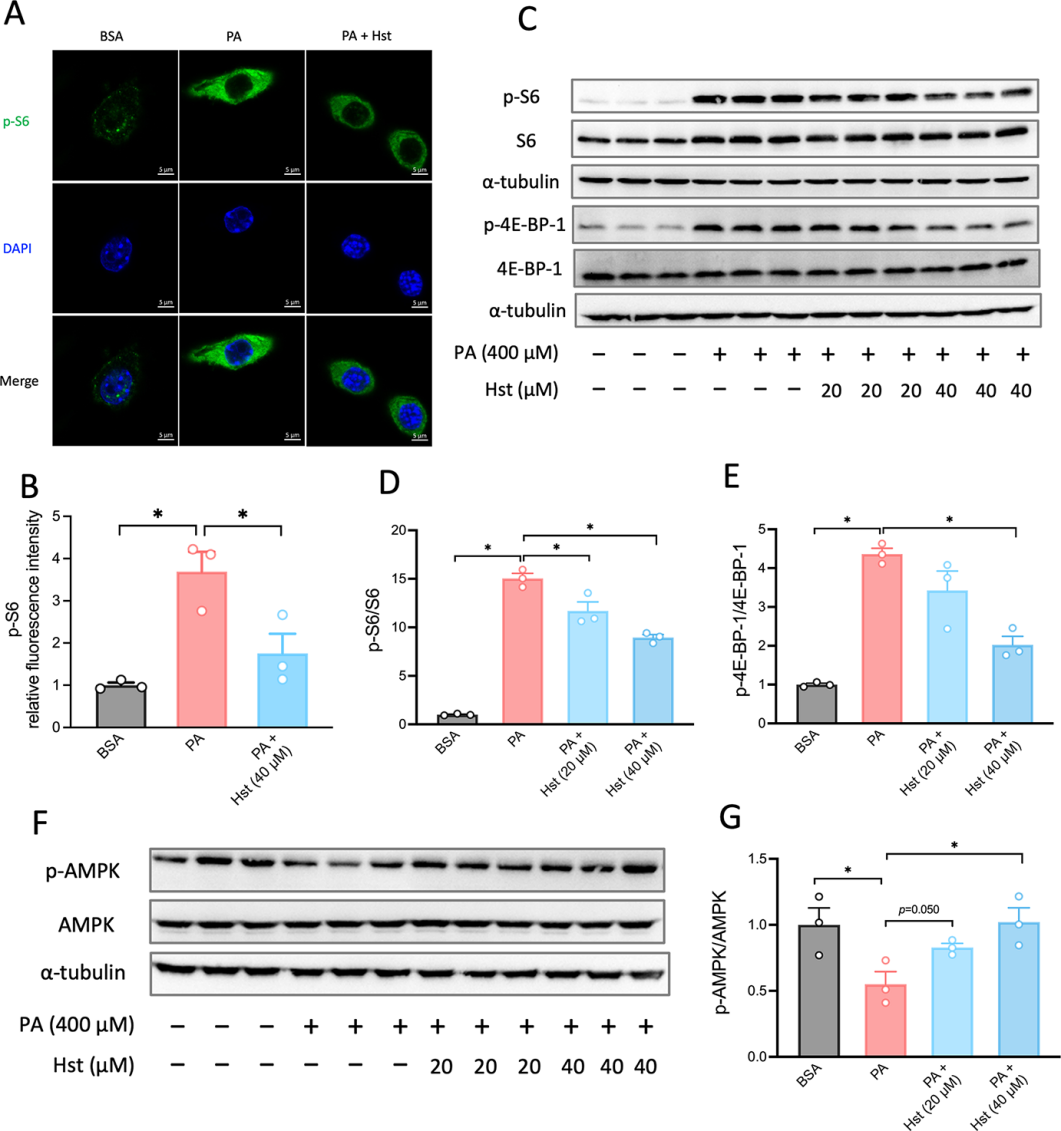
Hesperetin impaired palmitic acid-induced mTORC1 activation and stimulated AMPK activity. HepG2 cells were pretreated with hesperetin (20 μM and 40 μM) for 4 h, followed by incubation of palmitic acid (400 μM) for 10 h. (A) Immunofluorescence microscopy captions of HepG2 cells. Cells were stained against p-S6 (green) and DAPI (blue). (B) Quantification of the intensity of p-S6 as shown in (A). (C) Immunoblot analysis of mTORC1 activity markers (S6 and 4E-BP-1 phosphorylation) in HepG2 cells. (D and E) Quantification of phosphorylation levels of S6 and 4E-BP-1 as shown in (C). (F) Immunoblot analysis of AMPK phosphorylation in HepG2 cells. (G) Quantification of phosphorylation levels of AMPK in (F). All data are presented as the mean ± SEM (*n* = 3). Two-tailed unpaired Student’s test was used to calculate statistical significance. *p < 0.05. Scar bars are 5 μm in (A).

**Figure 6:**
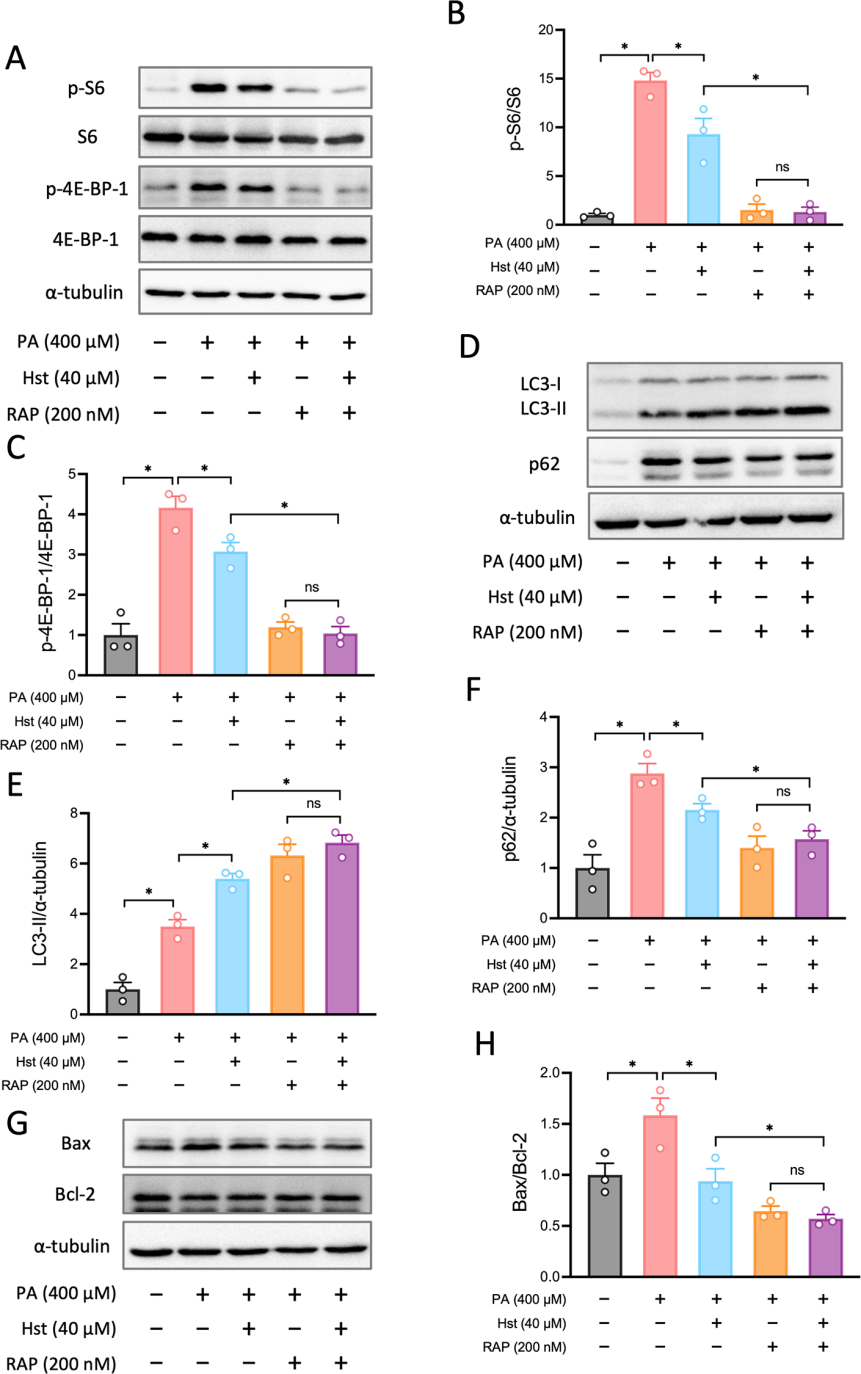
Hesperetin prevented palmitic acid-driven autophagy disruption and apoptosis through mTORC1 signaling. HepG2 cells were pretreated with hesperetin (40 μM) or RAP (200 nM) for 4 h, then incubated with palmitic acid (400 μM) for 10 h. (A) Representative blots of mTORC1 activity markers (S6 and 4E-BP-1 phosphorylation). (B and C) Quantification of phosphorylation levels of S6 and 4E-BP-1 as presented in (A). (D) Representative blots of autophagy markers (LC3I/II and p62). (E and F) Quantification of protein expression as presented in (D). (G) Representative blots of apoptosis markers (Bax and Bcl-2). (H) Quantification of the ration of Bax to Bcl-2 as presented in (G). All data are presented as the mean ± SEM (*n* = 3). Two-tailed unpaired Student’s test was used to calculate statistical significance. *p < 0.05.

### Hst Diminished Abnormal Glutamine Metabolism

It has been proposed that aberrant glutamine metabolism is linked to hepatocyte cell injury and increased hepatotoxicity.^6^ To uncover the potential effect of Hst on glutamine metabolism, we performed metabolomics analysis using GC-MS. We noticed a decrease in intracellular glutamate and α-KG levels, both glutaminolysis intermediates, following incubation of Hst compared to only PA-exposed HepG2 cells (Figure 7A, B). In a similar manner, correlating with glutamate and α-KG levels, reduced total intracellular pools of TCA intermediates, including succinate, fumarate, and malate, as well as pyruvate, were observed upon addition of Hst to PA-exposed HepG2 cells (Figure 7C). These results confirmed the reduction of glutamine channeling into the TCA cycle through α-KG. To gather more detailed information on this metabolic remodeling affected by Hst treatment, we conducted pulsed stable labeling studies to trace the intracellular fate of glutamine carbons in HepG2 cells using U-[^13^C]-glutamine incubation (Figure 7D). As expected, Hst incubation significantly lessened the incorporation of glutamine-derived carbons into glutamate and α-KG in PA-treated HepG2 cells (Figure 7E, F). In keeping with these metabolite levels, our results showed that less ^13^C-glutamine was incorporated into citrate and malate in Hst-treated HepG2 cells with respect to PA treatment alone (Figure 7G, H). These results confirmed that Hst mainly hampered the TCA cycle flux and inhibited the conversion of glutamine to glutamate and α-KG in PA-treated HepG2 cells.

**Figure 7:**
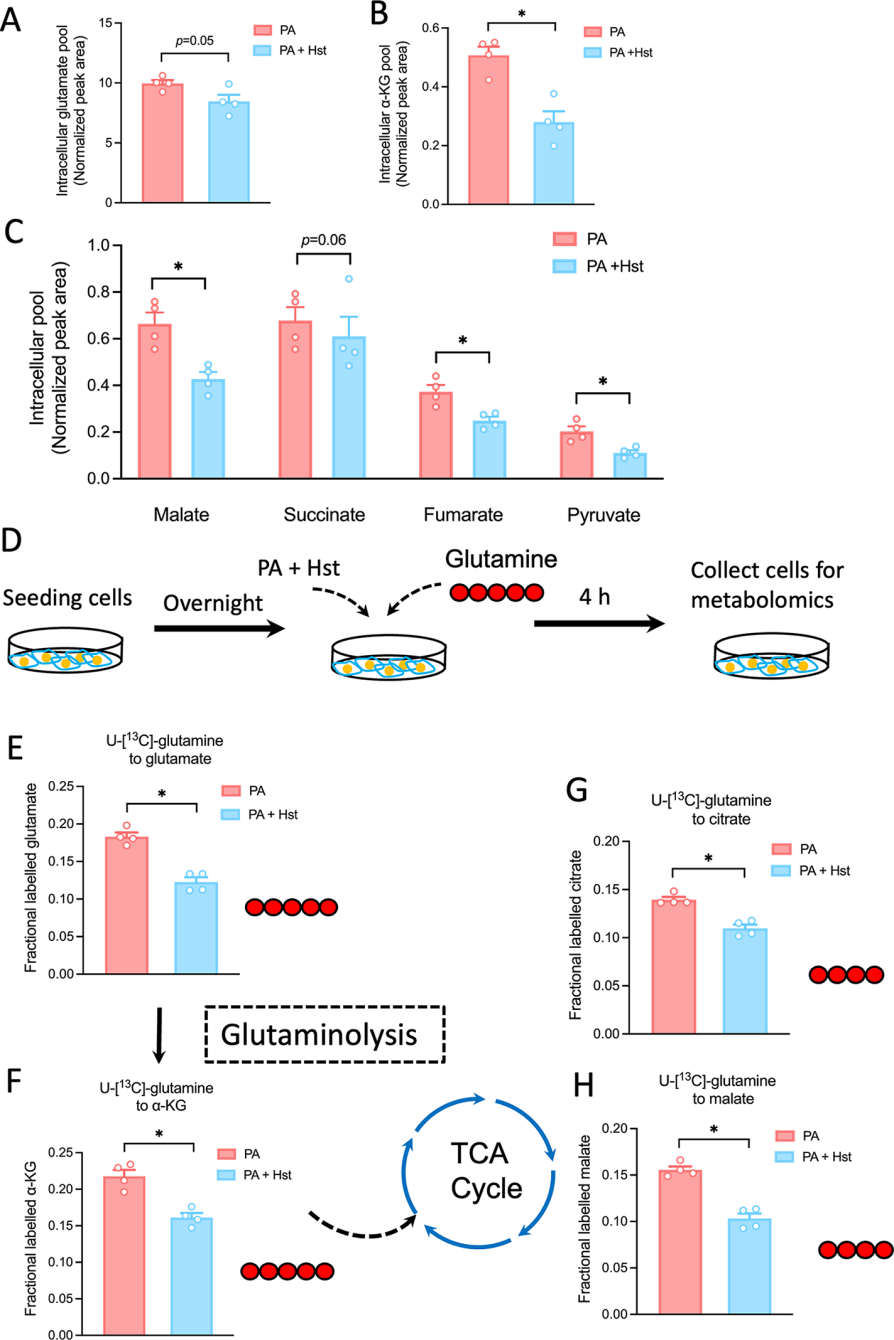
Hesperetin restored palmitic acid-disrupted glutaminolysis and TCA cycle. HepG2 cells were pretreated with palmitic acid (400 μM) for 4 h in the presence or the absence of hesperetin (40 μM) for another 4 h. Intracellular pool levels of glutamate (A) α-KG (B) and TCA cycle-related metabolites (C). (D) Schematic illustration of indicated treatments and U-^13^C-glutamine tracing in HepG2 cells. ^13^C-labeling of glutamate (E), α-KG (F), citrate (G), and malate (H) in HepG2 cells. All data are presented as the mean ± SEM (*n* = 4). Two-tailed unpaired Student’s test was used to calculate statistical significance. *p < 0.05.

### Hst Suppressed PA-Driven mTORC1 Activity and Apoptosis via the Regulation of Glutaminolytic α-KG Production

Considering that α-KG is the end product of glutaminolysis and is known to activate mTORC1, we hypothesized that Hst might impair glutaminolysis-mediated mTORC1 activation, thus decreasing cell apoptosis in PA-treated HepG2 cells. To tackle this hypothesis, we employed a cell-permeable derivative of α-KG, dimethyl-α-ketoglutarate (DMKG). As expected, addition of DMKG to PA-exposed HepG2 cells abolished the inhibitory effects of Hst on phosphorylation of S6 (Figure 8A, B), suggesting that the repression of glutaminolysis and α-KG production correlated with the effects of Hst on the PA-activated mTORC1 complex. Prompted by this, we evaluated whether glutaminolysis-generated α-KG was responsible for Hst-mediated induction of autophagy and reduction of cell apoptosis. As shown in Figure 8C, D, reduced LC3-II was detected in Hst-incubated HepG2 cells exposed to PA in the presence of DMKG. Next, we determined the extent to which the addition of DMKG contributes to Hst-mediated suppression of cell apoptosis. We found that DMKG abolished the ability of Hst to further affect apoptosis-related protein expression, such as the levels of caspase-3, Cyt *C*, Bax, and Bcl-2 (Figure 8E–H). Collectively, these results indicated that Hst impeded glutaminolysis-induced α-KG generation in PA-exposed HepG2 cells, halting mTORC1 complex activation and subsequently stimulating impaired autophagic flux, which ultimately prevented cell apoptosis.

**Figure 8:**
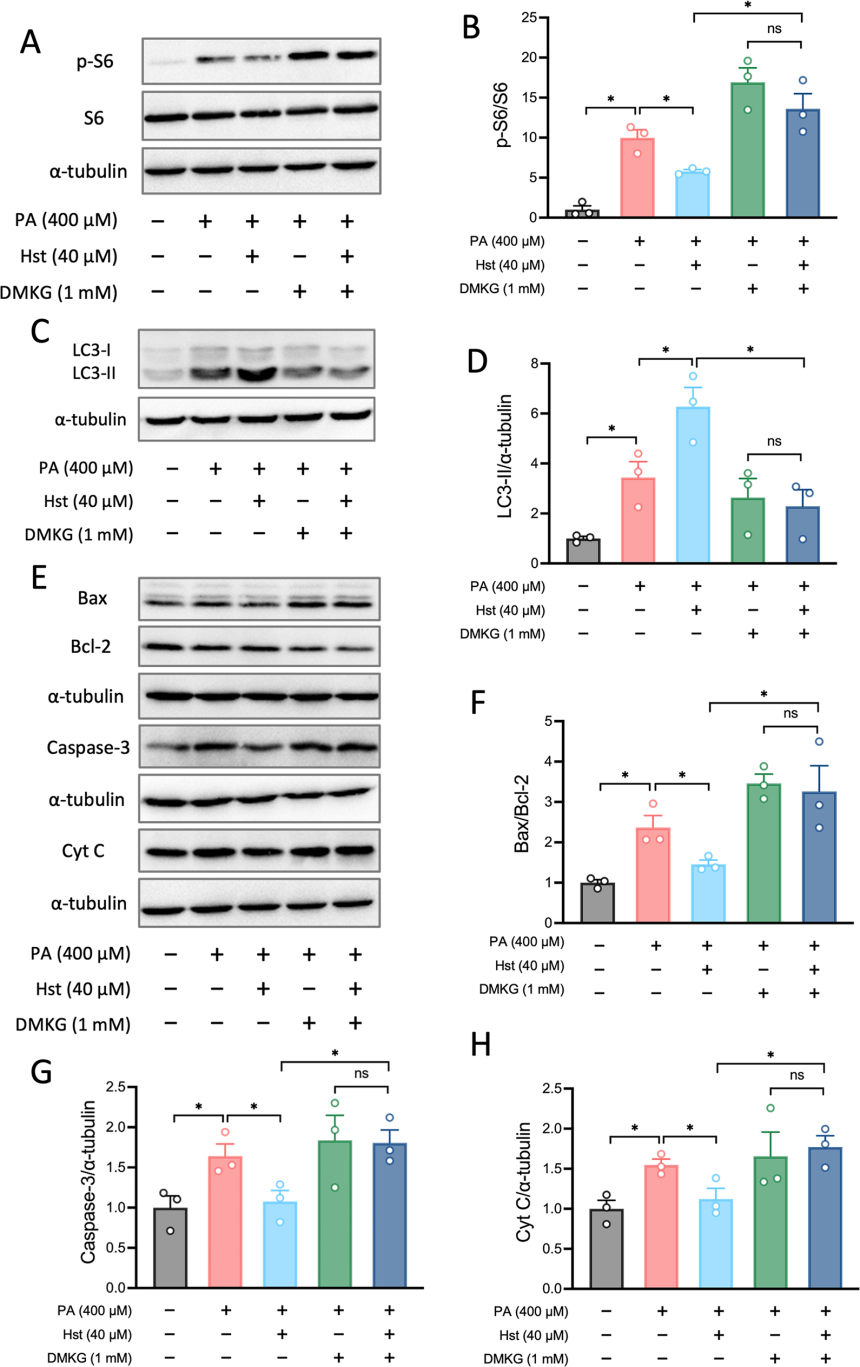
Hesperetin regulated palmitic acid-disrupted mTORC1 activity, autophagy, and diminished apoptosis via modulation of α-ketoglutarate production. HepG2 cells were preincubated with hesperetin (40 μM) or DMKG (1 mM) for 4 h, then stimulated with palmitic acid (400 μM) for 10 h. (A) Representative blots of mTORC1 activity markers (S6 phosphorylation). (B) Quantification of phosphorylation levels of S6 as presented in (A). (C) Representative blots of autophagy markers (LC3I/II). D, Quantification of protein expression as presented in (C). (E) Representative blots of pro-apoptotic markers (Bax, cleaved Caspase-3 and Cytochrome *C*) and antiapoptotic marker (Bcl-2). (F–H) Quantification of protein expression as shown in (E). All data are presented as the mean ± SEM (*n* = 3). Two-tailed unpaired Student’s test was used to calculate statistical significance. *p < 0.05.

### Hst Attenuated PA-Induced Apoptosis Associated with the Inhibition of Glutaminolysis and mTORC1 Signaling

So far, our results have suggested that glutaminolysis-derived α-KG was responsible for mTORC1 activation and subsequent autophagy suppression and enhancement of apoptosis. To assess the possible role that glutaminolysis plays in the modulation of Hst in response to PA treatment, we employed the inhibitor BPTES to pharmacologically inhibit GLS1, the main regulator enzyme of glutaminolysis. As shown in Figure 9A–C, GLS1 inhibition promoted the reduction of phosphorylation of S6 and 4E-BP-1 mediated by Hst in PA-incubated HepG2 cells. Likewise, glutaminolysis inhibition using BPTES strengthened the capacity of Hst to activate autophagy, as determined by the further elevated level of LC3-II and decreased level of autophagic endogenous marker p62 expression (Figure 9D–F). In a similar manner, BPTES treatment prevented the apoptosis stimulated by PA to a greater extent than HepG2 cells incubated with Hst, as evidenced by a lesser ratio of Bax/Bcl-2, caspase-3, and Cyt *C* protein expression (Figure 9G–J). These data indicated that the ability of Hst to prevent apoptosis and reactivate autophagy relies on its inhibition of glutaminolysis and mTORC1 activity in PA-stimulated HepG2 cells.

**Figure 9:**
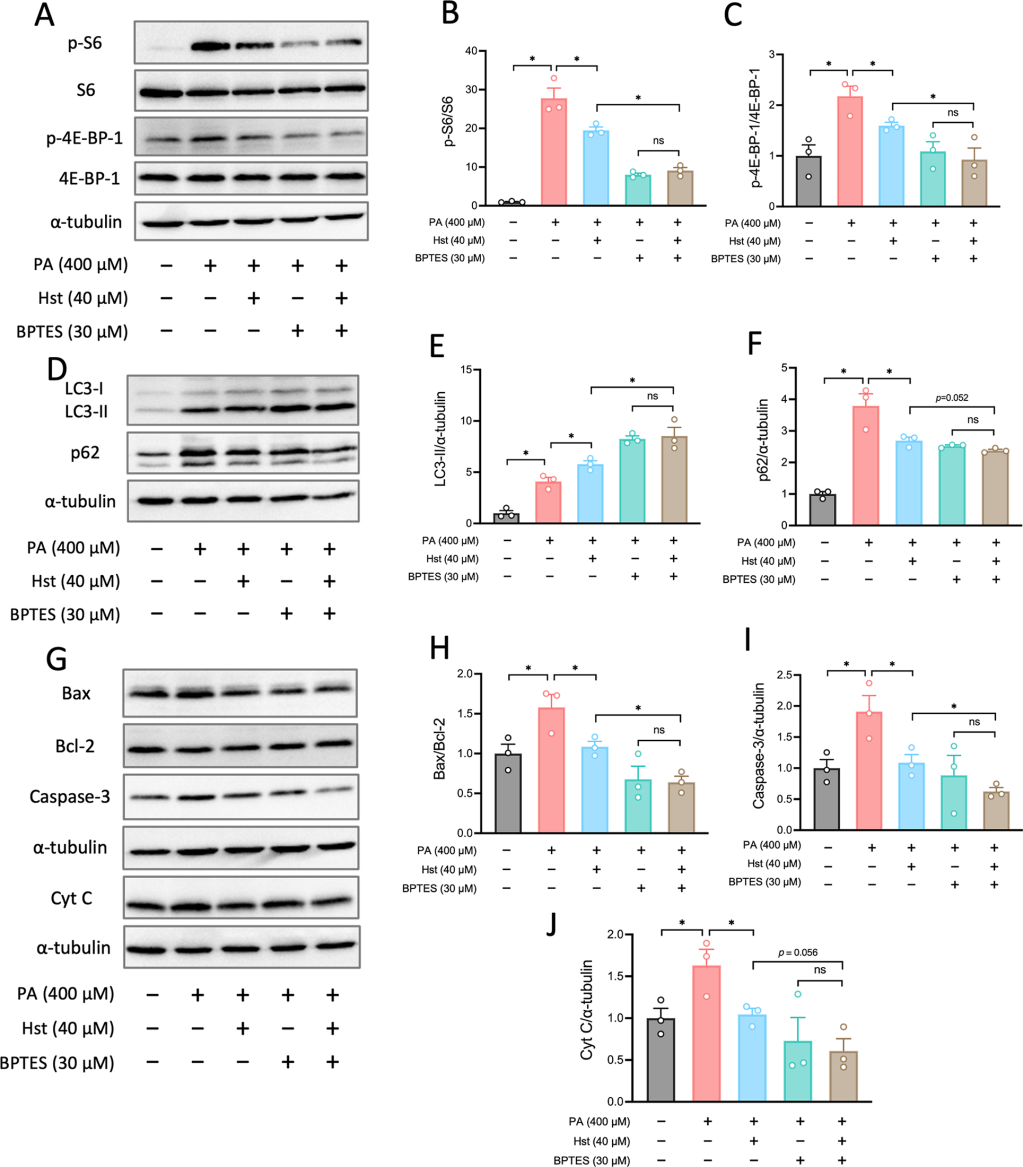
Hesperetin inhibited mTORC1 signaling, induced autophagy and reduced apoptosis involving inhibition of glutaminolysis. HepG2 cells were preincubated with hesperetin (40 μM) or BPTES (30 μM) for 4 h, then stimulated with palmitic acid (400 μM) for 10 h. (A) Representative blots of mTORC1 activity markers (S6 and 4E-BP-1 phosphorylation). (B and C) Quantification of phosphorylation levels of S6 and 4E-BP-1 as presented in (A). (D) Representative blots of autophagy markers (LC3I/II and p62). (E and F) Quantification of protein expression as presented in (D). (G) Representative blots of pro-apoptotic markers (Bax, cleaved Caspase-3, and Cytochrome *C*) and antiapoptotic marker (Bcl-2). (H–J) Quantification of protein expression as shown in (G). All data are presented as the mean ± SEM (*n* = 3). Two-tailed unpaired Student’s test was used to calculate statistical significance. *p < 0.05.

## Discussion

Hst belongs to the flavanone subclass of flavonoid, sharing similar structures and functional relationships with other flavanone glycosides, such as hesperidin (Hsd) and naringenin.^43,44^ These glycosylated precursors undergo hydrolysis in the gut, releasing their aglycone forms.^32,44^ The shared flavanone backbone, along with different hydroxylation patterns, contributes to their similar biological effects, including antioxidant, anti-inflammatory, and insulin-sensitizing properties.^45–47^ Driven by these properties, such compounds have attracted growing interest for their therapeutic potential in a wide range of diseases, including cardiovascular conditions,^48^ neurological disorders,^49^ and liver-related pathologies.^18,31^ In particular, the aglycone form, Hst, exhibits greater membrane permeability and water solubility than its glycosylated precursor, Hsd—both of which are critical determinants of bioavailability.^32^ These enhanced properties allow for more efficient gastrointestinal absorption, resulting in greater systemic circulation and more effective delivery to target tissues.^32,44^ Of note, Hst can penetrate the blood–brain barrier, an advantage that holds promise for mitigating the progression of neurodegenerative diseases.^32^ In this work, we report molecular mechanisms to explain how Hst diminishes PA-induced hepatotoxicity. These mechanisms are associated with the Hst-mediated regulation of impaired autophagic flux and aberrant apoptotic signaling, both of which critically contribute to FFA-induced hepatotoxicity. Hst reinstates the dysregulated glutaminolysis and normalizes the aberrant α-KG production derived from this pathway, while simultaneously enhancing AMPK activation. These molecular events culminate in the attenuation of mTORC1 activity, which, in turn, promotes autophagic flux and decreased cellular apoptosis. In addition, the enhanced phosphorylation of AKT and Nrf2 plays a pivotal role in mediating the cytoprotective effects of Hst under lipotoxic conditions.

Clear evidence has demonstrated that PA overload leads to lipotoxicity, which is directly linked to the activation of the intrinsic apoptotic pathway.^3,5^ Bcl-2 family members regulate the intrinsic apoptotic pathway by mediating Bax-induced mitochondrial permeabilization and the subsequent release of cytochrome *C* (Cyt *C*) from the mitochondrial intermembrane space. This is followed by the activation of effector caspase-3 from its inactive precursor, procaspase-3. The resulting signaling cascade directly triggers extensive cellular demise and consequent organ dysfunction.^50^ Therefore, attenuation of intrinsic apoptosis may counteract lipotoxicity, offering a promising intervention for managing NAFLD progression.^4,51^ Previous investigations have shown that Hst attenuated diabetes,^46^ hepatotoxicity,^52^ and liver fibrosis through amelioration of the intrinsic pathway of apoptosis.^53^ This prompted us to investigate whether Hst could relieve lipotoxicity in a model of PA-treated HepG2 cells. Our data suggested that Hst decreased PA-induced lipotoxicity through a mechanism similar to that observed in other models, primarily by inhibiting intrinsic apoptotic signaling. This might explain, at least in part, its ability to directly prevent PA-induced cell death. Our results provided evidence for the possibility of the potential application of Hst in clinical NAFLD treatment.

It is well established that stimulation of autophagy is sufficient to prevent cell apoptosis and promote cell survival.^14^ Our work corroborated the protection of Hst against apoptosis, requiring the activation of autophagy. Treatment with CQ enhanced the Hst-induced increase in LC3-II levels and prevented Hst-mediated reduction in p62, further reflecting an enhanced autophagic flux caused by Hst. Likewise, previous evidence suggested that Hst modulated autophagy to diminish neuronal impairment,^54^ intestinal barrier injury,^55^ and liver toxicity.^56^ These effects may result from the autophagy-mediated clearance of misfolded proteins, dysfunctional mitochondria, and excess lipid droplets within cells. Generally, there are several mechanisms accounting for the stimulation of autophagy. Our findings revealed that Hst activated autophagy, correlating with the repression of mTORC1 activity, whose activation is known to downregulate autophagy and promote cell apoptosis. It has been reported elsewhere that the regulation of mTORC1 activity by Hst may underlie its ability to exert beneficial effects in various stress conditions.^57,58^ In addition, we showed that the antiapoptotic effects of Hst on PA-exposed HepG2 cells were potentiated by rapamycin. Accordingly, rapamycin was observed to improve the impact of Hst on PA-impaired autophagy. This is in line with studies stating mTORC1 as a negative regulator of autophagy,^13,15^ with Hst mediating the autophagic pathway through mTORC1 signaling. Furthermore, our results indicated that Hst-induced AMPK activation was responsible for the suppression of mTORC1 activity and the promotion of autophagy. This is consistent with established observations that Hst-activated AMPK is inversely correlated with mTORC1 activity and positively associated with autophagy induction.^57^ Based on the above evidence, we speculate that Hst may induce autophagy and inhibit apoptosis through downregulation of the upstream signaling cascade mTORC1 and upregulation of AMPK activity in the case of PA-induced lipotoxicity. These results supported the roles of mTORC1 and AMPK in maintaining cellular homeostasis and highlighted targeting the mTORC1 signaling pathway as a plausible strategy for preventing NAFLD-mediated lipotoxicity. Although our results suggest the involvement of AMPK and mTORC1, direct mechanistic evidence linking Hst-induced AMPK activation to mTORC1 inhibition is lacking. Further studies using pathway-specific inhibitors could help clarify this potential regulatory relationship.

To gain more insight into the mechanisms underpinning the protection of Hst against lipotoxicity, we assessed its effect on the phosphorylation of AKT and Nrf2. Active AKT phosphorylates FOXO1, thereby influencing downstream regulators that govern lipid and glucose metabolism.^19^ Previous studies have reported that AKT activation is compromised in both palmitic acid-treated HepG2 cells and the STZ-induced high-fat diet (HFD) murine model.^19,59^ In contrast, Hst was found to enhance AKT and Nrf2 phosphorylation levels to attenuate OA-induced oxidative stress and inflammation in HepG2 cells and HFD mice.^18^ Moreover, a recent report has demonstrated that Hst protects PC12 cells from H_2_O_2_-induced oxidative damage by activating the AKT.^60^ Consistent with these findings, our results suggested that the activation of AKT was integral to mediating the protective effects of Hst against PA-induced lipotoxicity. It is not surprising that Hst, as a phenolic compound, may enhance the phosphorylation of Nrf2, a key regulator of redox homeostasis. The activation of the Nrf2/HO-1 pathway underpins many of the beneficial actions attributed to Hst, conferring significant protection against oxidative stress and cellular apoptosis.^18,61^ While these findings raise the possibility that the Nrf2/HO-1 axis contributes to the cytoprotective actions of Hst. Future investigations employing Nrf2-specific inhibitors would be valuable to delineate the precise role of the Nrf2/HO-1 axis and its interplay with other signaling cascades, such as mTORC1 signaling, in mediating the beneficial effects of Hst.

Glutaminolysis has been connected to cancer cell proliferation.^62^ In recent years, a growing body of literature has illuminated the important role of glutaminolysis in liver disease, such as NASH, fibrosis, and cirrhosis.^28,29^ Liver cells are one of the main organs involved in glutaminolysis. Although the detailed mechanisms of aberrant glutamine metabolism in triggering hepatocyte damage are not fully clear, increasing evidence implicates that interventions targeting glutaminolysis could effectively alleviate hepatocellular stress and damage.^6,29^ In our study, through metabolomics analysis and stable isotope tracing assays, we showed that Hst treatment prevented PA-driven apoptosis, which is associated with the regulation of glutamine metabolism impairment. Inhibition of glutaminolysis by BPTES promoted cell survival in HepG2 cells upon PA incubation, as previously observed in primary hepatic cells incubated with PA and OA.^6,29^ It must be mentioned that Hst repressed the production of α-KG driven by PA, which accounts for the replenishment of carbon units for sustaining the TCA cycle anaplerosis.^24^ Hst-mediated inhibition of glutaminolysis diminished the flux of α-KG in the TCA cycle. This might be one reason to explain the restoration of the TCA cycle mediated by Hst in the context of lipotoxicity. Accordingly, the stable isotope labeling results further confirmed these observations. Intriguingly, taking into consideration the significance of α-KG in relation to mTORC1 activity, our findings link the beneficial effects of Hst on mTORC1 to the inhibition of glutaminolysis. Our findings further substantiated that Hst-mediated inhibition of mTORC1 activity and induction of autophagy protected cells from apoptosis and damage, a mechanism largely attributable to the action of α-KG, as demonstrated by the addition of DMKG. These data are in agreement with previous reports in which activation of glutaminolysis followed by increased α-KG sustains the mTORC1 activity.^14,63^ These results provided evidence for the involvement of glutaminolysis in the beneficial effects of Hst and further highlighted the importance of glutaminolysis in activating mTORC1 signaling and preserving homeostasis in conditions of PA overload, indicating that targeting glutaminolysis may be a potential strategy for attenuating PA-induced lipotoxicity.

In summary, our findings suggest that the protective effects of Hst against PA-induced apoptosis and impaired autophagy are mediated through the inhibition of aberrant glutaminolysis and mTORC1 activity, alongside the activation of AMPK, and the recovery of AKT and Nrf2 activation under lipotoxic conditions. These results highlight the potential of glutaminolysis as a therapeutic target to alleviate lipotoxicity and provide evidence of possible dietary interventions to prevent NAFLD and other metabolic disorders.

## Supplementary Material



## Notes

Figure S1: hesperetin activated palmitic acid-impaired Nrf2 and Akt activity, enhancing HO-1 levels; HepG2 cells preincubated with hesperetin (20 and 40 μM) for 4 h, then stimulated with or without palmitic acid (400 μM) for 10 h; (A, C and E) representative blots of HO-1, Nrf2, and Akt phosphorylation; (B, D, and F) quantification of HO-1, p-Nrf2, and p-AKT expression levels as presented in (A, C, and E); all data presented as the mean ± SEM (*n* = 3); and two-tailed unpaired Student’s test used to calculate statistical significance, **p* < 0.05 (PDF)
