# Supplementary material for: Hesperetin Protects from Palmitic-Acid-Induced Lipotoxicity through the Inhibition of Glutaminolysis, mTORC1 Signaling, and Limited Apoptosis
Source: J Agric Food Chem. 2025 Aug 21;73(35):21932–46. doi: 10.1021/acs.jafc.5c05570 (PMC12412169; doi:10.1021/acs.jafc.5c05570)
Supplement: Supplementary file 1 [file jf5c05570_si_001.pdf]

Supporting Informmation

Hesperetin protects from palmitic acid-induced lipotoxicity through inhibition of glutaminolysis, mTORC1 signaling and limited apoptosis

Wan Li[1,2], Zhengnan Cai[1,2], Florian Schindler[1,3], Martin Brenner[1,2,3], Christian Winter[4], Bianca Stiller[5,6], Petra Heffeter[5], Wolfram Weckwerth[1,7,8]*

*Email: wolfram.weckwerth@univie.ac.at

[1]Molecular Systems Biology (MOSYS), Department of Functional and Evolutionary Ecology, University of Vienna, Vienna, 1030, Austria

[2]Vienna Doctoral School of Ecology and Evolution, University of Vienna, Vienna, 1030, Austria

[3]Vienna Doctoral School of Pharmaceutical, Nutritional and Sports Sciences, University of Vienna, Vienna, 1090, Austria

[4]Marine Biology, Department of Functional and Evolutionary Ecology, University of Vienna, Vienna, 1030, Austria

[5]Center for Cancer Research and Comprehensive Cancer Center, Medical University of Vienna, Vienna, 1090, Austria

[6]Research Cluster "Translational Cancer Therapy Research", University of Vienna,

Vienna, 1090, Austria

[7]Vienna Metabolomics Center (VIME), University of Vienna, Vienna, 1030, Austria

[8]Health in Society Research Network, University of Vienna, Vienna, 1030, Austria

Figure S1

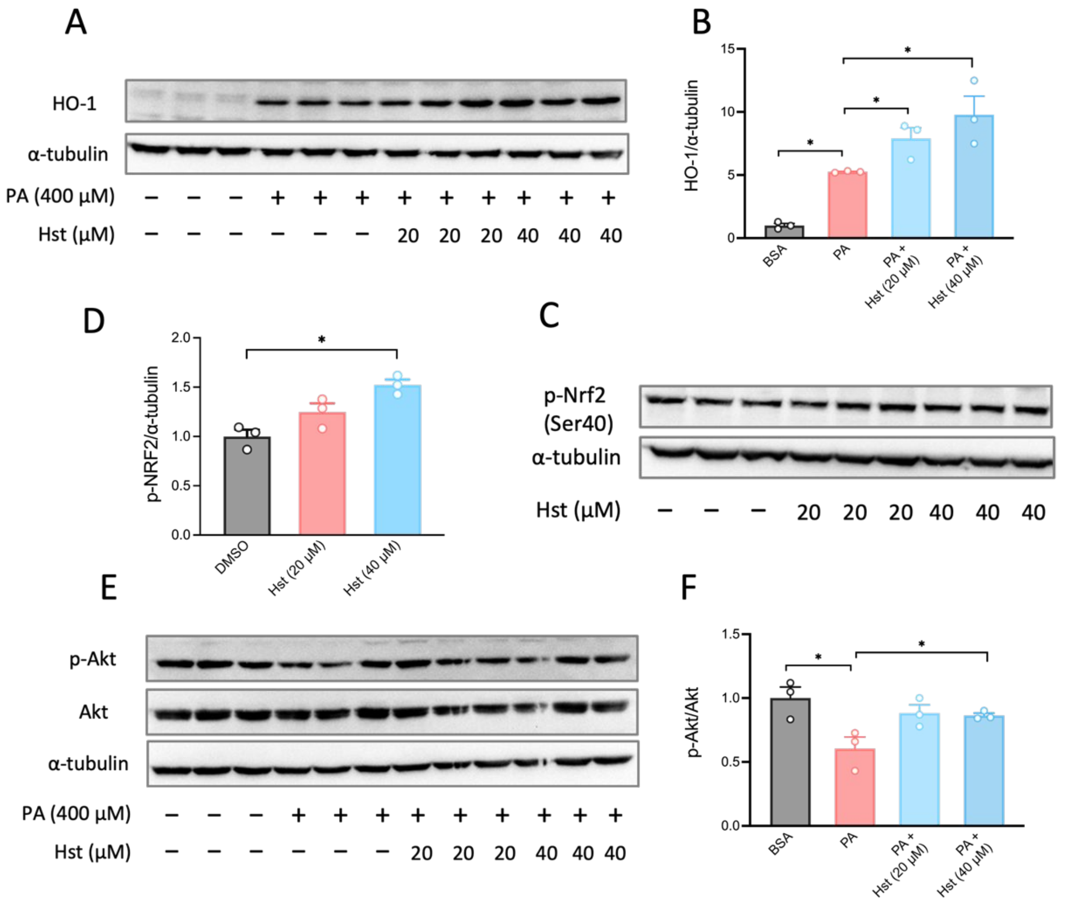

Figure S1. Hesperetin activated palmitic acid-impaired Nrf2 and Akt activity, enhancing HO-1 levels. HepG2 cells were preincubated with hesperetin (20 μM and 40 μM) for 4 h, then stimulated with or without palmitic acid (400 μM) for 10 h. A, C and E, Representative blots of HO-1, Nrf2 and Akt phosphorylation. B, D and F, Quantification of HO-1, p-Nrf2 and p-AKT expression levels as presented in (A, C and E). All data are presented as the mean ± SEM. (n=3). Two-tailed unpaired Student's test was used to calculate statistical significance. *$p < 0.05$.
